# Growth factor and hypoxia-inducible factor alpha content in the retina of male Wistar rats in experimental diabetic retinopathy and the effect of cellular protein kinase blockade

**DOI:** 10.3389/fendo.2025.1643445

**Published:** 2025-10-15

**Authors:** K. O. Usenko, Olena Strubchevska, S. O. Rykov, M. S. Babenko, Kateryna Strubchevska, Oleksandra Kozyk, S. V. Ziablitsev, Marko Kozyk

**Affiliations:** ^1^ Nacional’nij medicnij universitet imeni O Bohomol’ca, Kyiv, Ukraine; ^2^ Uniwersytet Jagiellonski w Krakowie Collegium Medicum, Kraków, Poland; ^3^ Corewell Health William Beaumont University Hospital, Royal Oak, MI, United States; ^4^ Willamette University, Portland, OR, United States

**Keywords:** diabetes mellitus, diabetic retinopathy, VEGF, HIF-1α, western blot, immunohistochemistry

## Abstract

**Background:**

Diabetic retinopathy (DR) is a leading cause of vision loss in patients with diabetes mellitus (DM). Hypoxia-driven overexpression of vascular endothelial growth factor (VEGF) and hypoxia-inducible factor-1α (HIF-1α) is central to diabetic retinopathy (DR) pathogenesis. The use of cellular protein kinase inhibitors is a promising approach for correcting pathological changes in DR.

**Objective:**

To determine the effect of pharmacological blockade of cellular protein kinases with sorafenib on the expression of VEGF and HIF-1α in the retina in experimental diabetic retinopathy.

**Material and methods:**

Diabetes mellitus (DM) was induced in male rats by administration of streptozotocin (50 mg/kg). Animals were divided into three groups: in group 1 (n=15) rapid-acting insulin at a dose of 30 U was injected intraperitoneally, in group 2 (n=15) insulin was combined with sorafenib (*per os* 200 mg), and in the control group (n=15) no treatment of hyperglycemia was performed. 5 animals were used to obtain baseline data (intact). The drugs were administered every other day, starting from day 7 after streptozotocin injection, for 3 months. Immunohistochemical studies were performed using monoclonal mouse antibodies against VEGF. Sections were additionally stained with hematoxylin. The content of VEGF and HIF-1α in retinal tissue lysates was determined by Western blotting. Membranes with proteins were incubated with monoclonal antibodies against VEGF and HIF-1α. After the initial incubation, the membranes were washed and treated with anti-species secondary antibodies conjugated to horseradish peroxidase. Statistica 10 software was used for statistical analysis. Descriptive statistics were calculated, including means and their standard errors. Sample averages were compared using analysis of variance (ANOVA), with p-values less than 0.05 considered statistically significant.

**Results:**

Under the conditions of experimental DR, the content of VEGF in retinal tissues increased significantly and after 3 months of observation increased 6,8-fold for the dimeric form and 27.1-fold for the monomeric form (p<0,05) compared to intact animals. Under the same conditions, the level of HIF-1α was also significantly increased (39.6-fold; p<0.05). When insulin was administered, the content of VEGF fractions in the retina decreased by an average of 1,4-1,5 times (p<0,05), and the heterogeneity of the response to its administration was observed. The use of sorafenib with insulin in all cases blocked the increase in VEGF content caused by DR. Insulin administration reduced HIF-1α levels by 1,4-fold (p<0,05) compared to the control, whereas combined sorafenib and insulin treatment reduced HIF-1α expression to undetectable levels. Immunohistochemical examination revealed a progressive increase in the intensity of VEGF-positive staining in the retina during experimental DR, as well as the development of its degenerative changes - edema, ischemia, pathological angiogenesis, neurodegeneration, and disruption of retinal layer organization. The use of insulin did not cause changes in the retinal pattern, whereas the combined use of sorafenib and insulin prevented the development of both morphological signs of DR and an increase in the intensity of VEGF-positive staining.

**Conclusion:**

The significance of VEGF and HIF-1α upregulation in the pathogenesis of DR and the effectiveness of their correction by pharmacological blockade of cellular protein kinases with sorafenib have been established.

## Introduction

Diabetic retinopathy (DR) is one of the leading causes of vision loss in patients with diabetes mellitus and represents a significant challenge for healthcare systems (1–3). The central pathobiological driver of early vascular and neuroretinal damage in DR is hypoxia-induced upregulation of the HIF1α–VEGF axis (hypoxia-inducible factor alpha – vascular endothelial growth factor), which promotes angiogenesis and increases blood-retinal barrier (BRB) permeability, leading to edema, microangiopathy, and progressive neurodegeneration (4–12). Under normoxia, HIF1α is rapidly degraded via PHD/pVHL (prolyl-4-hydroxylase domain/Von Hippel–Lindau protein)-dependent mechanisms; however, under hypoxic conditions it becomes stabilized and transcriptionally upregulates VEGF and other adaptive genes (5–7, 13, 14). Upregulation of VEGFR2 modulates endothelial survival, proliferation, and adhesive contacts (VE-cadherin), directly affecting BRB permeability and edema formation (9–11). Thus, the HIF1α–VEGF axis remains the most validated target for preventing early retinal damage in DR (4–7, 11).

Under physiological conditions, VEGF is predominantly localized in the ganglion cell layer and retinal pigment epithelium; in DR, its expression expands to Müller cells, astrocytes, and ischemic regions, correlating with BRB dysfunction and neurovascular unit remodeling (15–20). These changes underlie the structural and functional visual impairments; thus, targeting hypoxia–angiogenesis signaling nodes is a logical strategy for modifying DR progression (11). Current therapeutic approaches focus mainly on post-receptor VEGF blockade (intravitreal anti-VEGF agents), which effectively reduce edema and neovascularization but have limitations: variability of response, injection burden, and incomplete control of upstream hypoxic signaling (9–12, 21–23). An additional clinical challenge is the effect of insulin therapy: intensive glycemic normalization may transiently exacerbate BRB disruption via HIF1α/VEGF-dependent mechanisms, and insulin use is associated with a risk of macular edema — a context that underscores the need for adjuvant strategies beyond glycemic control (24–26). Conceptually, multikinase inhibition is attractive as it acts higher in the regulatory cascade (interrupting MAPK/ERK signaling and HIF1α stabilization) and at the level of VEGFR/PDGFR, potentially complementing anti-VEGF therapy (9–12, 21–23).

Sorafenib is a multikinase inhibitor that targets RAF (cRAF [cellular RAF serine/threonine-protein kinase], BRAF [B-RAF proto-oncogene serine/threonine-protein kinase]) and angiogenic receptors (VEGFR1/2/3, PDGFRβ), as well as other tyrosine kinases sustaining angiogenesis (27–30). Preclinical studies have shown that sorafenib reduces HIF1α/VEGF levels, microvascular density, and cellular invasiveness; mechanistically, these effects are attributed to blockade of the MAPK/ERK axis, disruption of nuclear translocation/binding of HIF1α to HRE (hypoxia response element), and inhibition of HIF1α translation via suppression of the mTOR/RPS6KB1/4EBP1 pathway (mammalian target of rapamycin/ribosomal protein S6 kinase B1/eukaryotic translation initiation factor 4E-binding protein 1) (28, 31). Concomitant inhibition of VEGFR2/3 and PDGFRβ may attenuate permeability, endothelial proliferation, and pathological neovascularization—events critical for DR progression (9–12, 21–23, 27, 28, 30, 31).

Despite this mechanistic rationale, a knowledge gap remains: whether sorafenib-mediated multikinase blockade can suppress HIF1α/VEGF specifically in the retina under diabetic conditions and preserve its architecture over time compared with standard glycemic correction, and to what extent such a strategy conceptually complements anti-VEGF therapy through both upstream and receptor-level actions (9–12, 21–23, 27, 28, 30, 31). To address this question, we employed a streptozotocin (STZ)-induced diabetes model in Wistar rats and compared standard insulin therapy with a combination of insulin plus sorafenib. Quantitative evaluation of HIF1α/VEGF was performed by Western blotting, while VEGF localization was assessed by immunohistochemistry at predefined time points. This design allowed integration of quantitative biomarkers with early morphological hallmarks of DR and enabled us to test the hypothesis of targeted modulation of the HIF1α–VEGF axis.

Thus, we hypothesized that sorafenib, as a multikinase inhibitor, would reduce HIF1α/VEGF expression in the retina and mitigate early DR-like damage beyond the effect of insulin therapy, thereby potentially complementing anti-VEGF agents by acting upstream at the RAF/ERK cascade and at the level of VEGFR/PDGFR (9–12, 21–23, 27, 28, 30, 31, 86, 87). At the same time, we acknowledge the translational limitations of systemic multikinase therapy in DR and aim for further evaluation of locally targeted or pathway-selective strategies for drug delivery/target inhibition (11). Notably, adverse effects of systemic sorafenib therapy in humans include diarrhea, arterial hypertension, skin reactions, thyroid dysfunction, and proteinuria (32, 33), as well as peripheral neuropathy, the pain of which is unrelieved by standard analgesics and frequently leads to treatment discontinuation (34). This positioning is consistent with contemporary clinical approaches and emphasizes a rational combination of glycemic control with targeted modification of hypoxia–angiogenesis pathways in DR.

The aim of this study was to determine the retinal content of vascular endothelial growth factor (VEGF) and hypoxia-inducible factor-1α (HIF-1α) in experimental diabetic retinopathy and to assess the effect of pharmacological blockade of cellular protein kinases with sorafenib on these parameters.

## Materials and methods

To address the aim of the study, we used a streptozotocin (STZ)-induced diabetes model in Wistar rats and compared standard insulin therapy with a combination of insulin + sorafenib. Quantitative assessment of HIF1α/VEGF was performed using Western blotting, and VEGF localization was evaluated by immunohistochemistry at predefined time points. This design allowed us to integrate quantitative biomarkers with early morphological features of DR and to test the hypothesis of targeted modulation of the HIF1α–VEGF axis.

All experimental procedures were carried out in accordance with the norms and principles of the European Convention for the Protection of Vertebrate Animals Used for Experimental and Other Scientific Purposes (Strasbourg, 1986), the Council Directive 86/609/EEC (1986), the Law of Ukraine No. 3447-IV “On Protection of Animals from Cruelty,” the general ethical principles of animal experimentation adopted by the First National Congress of Bioethics of Ukraine (2001), and the expert approval of the Commission on Bioethical Expertise and Ethics of Scientific Research at the Bogomolets National Medical University.

The study involved 50 three-month-old male Wistar rats weighing 140–160 g. Experimental diabetes mellitus (DM) was induced in 45 animals by a single intraperitoneal injection of streptozotocin (50 mg/kg; Sigma-Aldrich, Co, China) dissolved in cold 0.1 M citrate buffer (pH 4.5). Five animals were used to obtain baseline (intact) values. Animals were fasted for 16 h before injection and received 5% glucose solution for 24 h afterwards. Blood glucose levels were subsequently monitored every three days using a glucometer and disposable test strips (ACCU-Check Instant, Roche, Mannheim, Germany) from tail vein blood samples collected in the fasting state. Three days after streptozotocin administration, blood glucose levels in treated rats were not lower than 15 mmol/L. Animals were observed for a total of three months.

In this study, we deliberately included only male rats to minimize hormonally driven variability in the results (estrogen-dependent regulation of VEGF/HIF1α, differences in DR pathogenesis between males and females) (35–38), and we plan a separate validation in females taking into account their cycle status. Under these conditions, comparing insulin versus insulin + sorafenib makes it possible to distinguish the contribution of glycemic control from upstream/receptor-level modulation of angiogenic signaling (24–26).

Seven days after induction, animals with persistent hyperglycemia (n = 45) were randomly assigned to three groups of 15 animals each using simple blinded randomization. The researchers performing the allocation (technical staff of the vivarium) were unaware of the experimental interventions and did not know which group would serve as the control or experimental group. Group 1 (control) remained untreated for hyperglycemia. Group 2 received short-acting insulin (Actrapid HM Penfill, Novo Nordisk A/S, Bagsværd, Denmark) intraperitoneally every other day at a dose of 30 U. Group 3 received the same insulin regimen as Group 2 and, in addition, were administered sorafenib (Cipla, India) orally once daily at a dose of 50 mg/kg, prepared ex tempore as a sachet solution. One tablet of the drug (200 mg) was homogenized in a porcelain mortar, dissolved in 20 ml of sterile physiological saline (pH 6.7), and immediately administered to the animal via a gavage needle at a dose of 0.5 ml per 100 g of body weight.

Animals were euthanized at 7 and 28 days, and at 3 months, with five animals from each group sacrificed via lethal thiopental injection (75 mg/kg). For morphological analysis, the eyes were fixed in 10% neutral formalin and embedded in paraffin. Serial histological sections 2–3 μm thick were prepared from the paraffin blocks using a rotary microtome HM 325 (Thermo Shandon, UK). Morphological examinations were performed by two independent teams of expert morphologists, who provided assessments for each specimen. The slides were numbered, but the morphologists were blinded to group allocation. The study was conducted at the Department of Morphology, National University of Health of Ukraine (Kyiv), under the supervision of Prof. Olena Dyadyk.

Immunohistochemical analysis was performed using monoclonal mouse antibodies against VEGF (Invitrogen, VEGF Monoclonal Antibody (VG1), VG1, Catalog #MA1-16629) at 1:200 dilution in 0.1% BSA, Thermo Fisher Scientific, Waltham, Massachusetts, USA) on adhesive Super Frost Plus slides (Menzel, Germany). High-temperature antigen retrieval was carried out using citrate buffer (pH 6.0) or EDTA buffer (pH 8.0), and detection was performed with the Master Polymer Plus Detection system (Master Diagnostica, Spain). Sections were additionally counterstained with hematoxylin. Microscopic examination and image archiving were conducted using light microscopes ZEISS (Germany) equipped with the Axio Imager.A2 imaging system at objective magnifications of 5×, 10×, 20×, and 40×, a binocular 1.5× attachment, and 10× ocular lenses, with ERc 5s cameras, as well as Carl Zeiss Primo Star and Axiocam 105 color cameras, and an Olympus BX 40 light microscope additionally equipped with a digital Olympus C3030-ADU camera and Olympus DP-Soft software.

The evaluation of staining intensity in cells was performed according to the recommendations of D. Dabbs (2021) using a visual analog scale (39). A score of 0 indicated no staining, 1 – weak, 2 – moderate, and 3 – strong staining intensity. The number of VEGF-positive ganglion cells with different staining intensities was counted using an object micrometer, and the results were calculated as the number of cells per mm² and expressed as a percentage.

The determination of VEGF and HIF-1α content in retinal tissue lysates was carried out by Western blotting at the Department of Enzyme Chemistry & Biochemistry, Palladin Institute of Biochemistry, Kyiv, Ukraine, under the supervision of Prof. Artem Tykhomyrov. Tissue samples were preserved in liquid nitrogen, minced, and homogenized in 50 mmol Tris-HCl buffer (pH 7.4) supplemented with phosphatase and protease inhibitors (Pierce Protease and Phosphatase Inhibitor, Thermo Scientific, USA, #A32961), followed by additional disruption using ultrasound. After centrifugation, the total protein concentration in the supernatant was determined spectrophotometrically (40). Electrophoresis was performed in an 8% polyacrylamide gel with sodium dodecyl sulfate (SDS-PAGE) (41) using a vertical gel electrophoresis chamber (Bio-Rad, USA). Sample stacking was performed at 30–35 V (15–18 mA), and separation at 45–50 V (30–35 mA). Proteins were transferred from the gel onto a nitrocellulose membrane by electroblotting using a buffer solution containing 0.025 mol Tris-HCl, 0.192 mol glycine, and 25% methanol (42). The membranes were blocked with 5% non-fat dry milk solution in phosphate-buffered saline (PBS), followed by incubation with monoclonal antibodies against VEGF (Invitrogen, USA, no. MA5-12184, mouse, 1:3,000 dilution) and HIF-1α (Sigma Aldrich, USA, no. HPA001275, rabbit, 1:2,500 dilution). Antibodies against β-actin (loading control, Invitrogen, USA, no. MA5-15739, mouse, 1:3,000 dilution) were used as a control for protein loading. After primary incubation, membranes were washed and treated with horseradish peroxidase-conjugated secondary antibodies (goat anti-rabbit or anti-mouse IgG, Invitrogen, USA, cat. nos. G-21234 and 31430, respectively, 1:8,000 dilution). Semiquantitative analysis was performed densitometrically using TotalLab software (TL120, Nonlinear Inc., USA). Background subtraction was performed using by TotalLab (TL120, Nonlinear Inc.), and signal intensities were validated to fall within the linear dynamic range of detection. To ensure reproducibility, each blot was quantified in duplicate, and technical replicates were included for key experimental conditions. Inter-blot variability was minimized by using consistent exposure times, identical antibody batches, and internal loading controls across all experiments. Protein molecular weights were determined by comparing their migration on the nitrocellulose membrane with stained markers of the PageRuler™ Plus Prestained Protein Ladder (Thermo Scientific, Lithuania, cat. no. 26619) in the 10–230 kDa range. The results of Western blot analysis of VEGF and HIF-1α content were expressed as percentages relative to the control optical density value of the corresponding polypeptide band (in arbitrary units) on the blots, normalized to actin content in each sample (VEGF/actin and HIF-1α/actin). During the laboratory examination, retinal samples were blinded by assigning random numbering, so that the researchers had no information about the group allocation of the samples.

For statistical analysis, EZR v. 1.68 software (a graphical user interface for R statistical software version 4.3.1, R Foundation for Statistical Computing, Vienna, Austria) was used. In this study, statistical comparisons were performed for the group assessment results of VEGF26/actin, VEGF45/actin, and HIF-1α/actin ratios in rat retinal tissue, as well as for the number of cells with different intensities of VEGF-positive staining in the inner nuclear layer. Data were recorded as median values (Me) and interquartile range (QI–QIII). The Shapiro–Wilk test was applied to assess normality. The Kruskal–Wallis test was used to determine differences among groups; when the test result was significant, *post hoc* pairwise comparisons were performed using Dunn’s test. Statistical experts conducted group comparisons in a blinded manner and had no information regarding group allocation.

## Results

**Figure 1:**
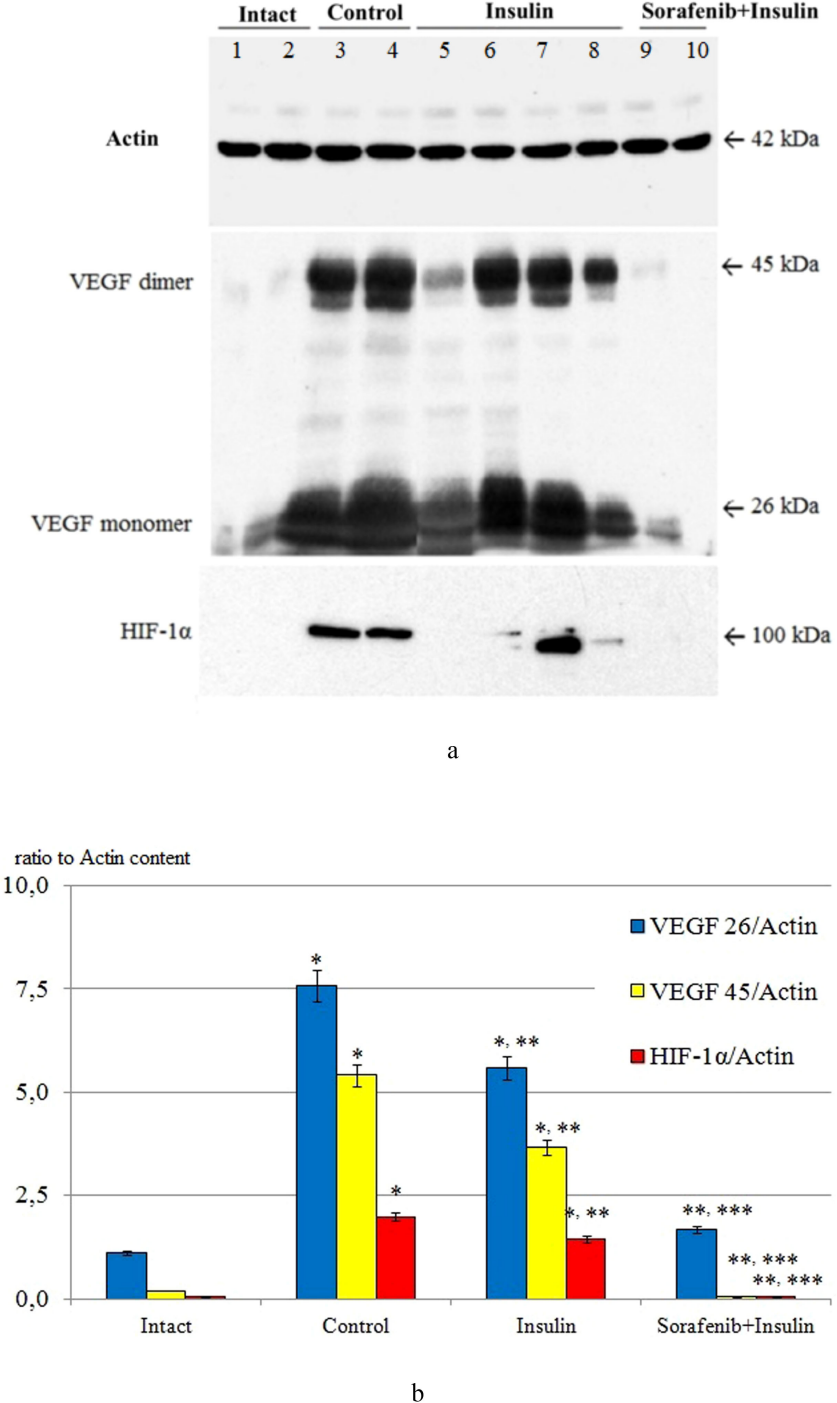
The ratio of VEGF 26/actin, VEGF 45/actin and HIF-1α/actin in the retinal tissue of intact animals (Intact; lanes 1, 2), after 3 months in the control group (Control; lanes 3, 4), the group with insulin administration (Insulin; lanes 5-8) and the group with combined administration of sorafenib with insulin (Sorafenib+Insulin; lanes 9, 10); **(A)** representative blotograms of actin, VEGF, and HIF-1α; **(B)** results of densitometric analysis of blotograms (ratio to actin content); *p<0,05 compared with intact; **p<0,05 compared with control; ***p<0,05 compared with insulin.

Under the conditions of experimental DR, the content of VEGF in retinal tissues increased significantly and after 3 months of observation compared with the initial level (intact animals) was increased (**Figure 1**) – for the dimeric form (precursor protein) by 6,8 times, for the monomeric form (active form) by 27,1 times (p<0,05 for both cases).

When insulin was administered, the content of VEGF fractions in the retina on average exceeded the initial data (see **Figure 1**) by 5.0 and 18.3 times, respectively (p < 0.05 for both cases). The difference compared with the control group was statistically significant (p < 0.05), indicating a certain effect of insulin on reducing diabetic retinal VEGF overexpression (1.4–1.5 times; p < 0.05). It should be noted that in 40% of animals, the VEGF content was significantly reduced (2.7-fold for the dimeric form and 1.7-fold for the monomeric form, respectively; p < 0.05 for both cases), while in the rest of the animals it did not differ from the control group. This fact pointed to the heterogeneity of the response of VEGF expression to insulin and requires separate discussion.

The use of sorafenib with insulin in all cases blocked the increase in VEGF content caused by diabetes (see **Figure 1**) – the content of this growth factor in the retina did not differ from the baseline data (p>0,05).

The increase in VEGF expression is closely related to the upregulation of the powerful transcription factor HIF-1α under hypoxic conditions (43).

Its content in retinal tissues was also studied by Western blotting (see **Figure 1**). The initial level of HIF-1α (intact animals) was below the detection level of the method, indicating a low level of expression in normal rat retinal tissues. After 3 months, in the control group, its level was significantly increased (39.6-fold compared with the densitometric optical density indices of samples from intact animals; p < 0.05), which, in our opinion, corresponded to the development of tissue hypoxia (44, 45).

Insulin administration decreased HIF-1α levels by 1.4-fold (p<0,05) compared to control group, whereas the combined administration of sorafenib and insulin was associated with the absence of HIF-1α expression (see **Figure 1**).

**Figure 2:**
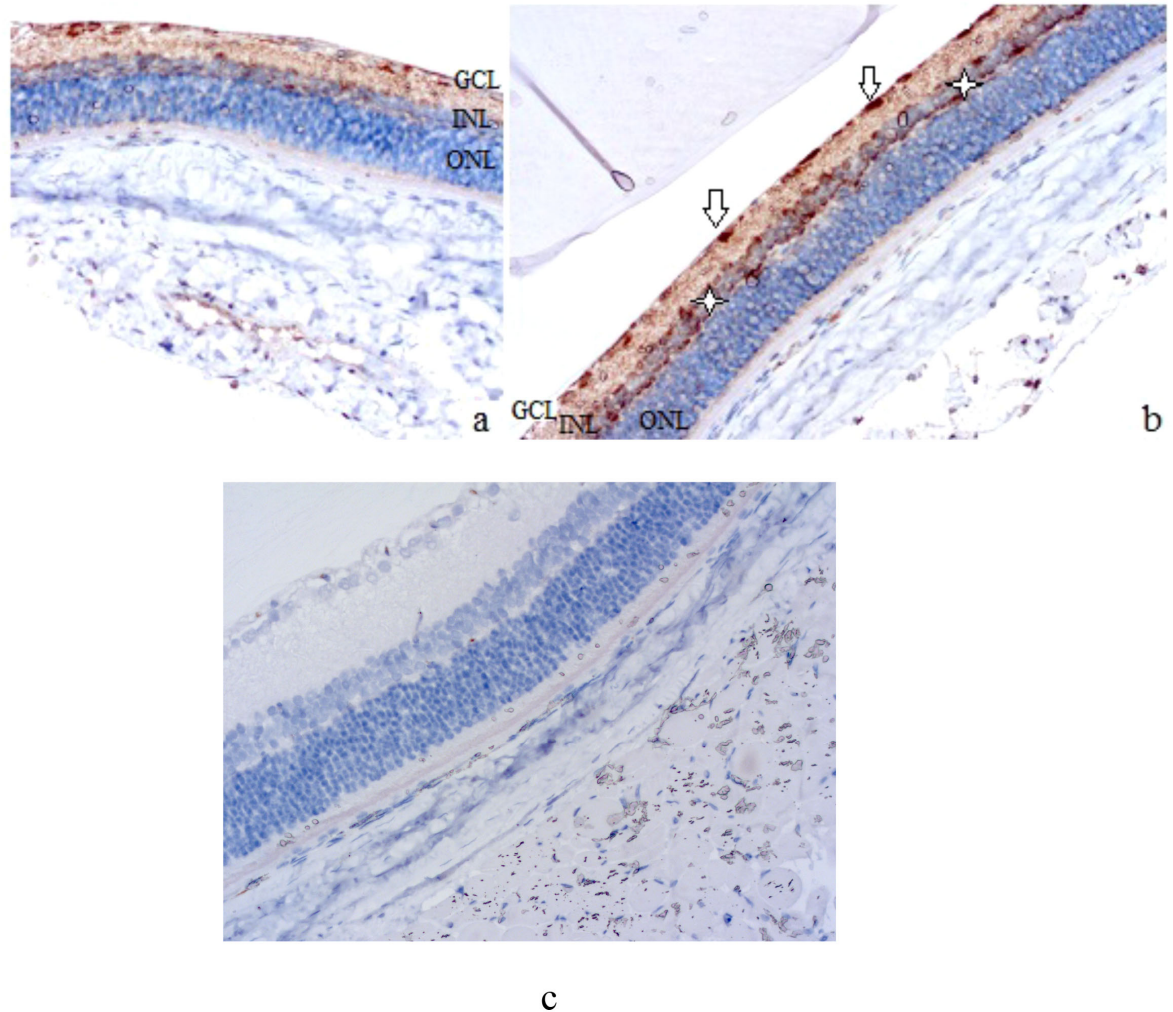
Rat retinal micropreparations (**A** – intact retina; **B** – day 7; **C** – day 7, negative control). Representative results of VEGF immunohistochemical staining, counterstained with hematoxylin; ×200. GCL, ganglion cell layer; INL, inner nuclear layer; ONL, outer nuclear layer. In **(A)** minimal VEGF-positive staining in the ganglion cell layer; in **(B)** white arrows indicate VEGF-positive ganglion cells, white asterisks indicate Müller cells at the periphery of the inner nuclear layer; **(C)** absence of specific staining.

**Figure 3:**
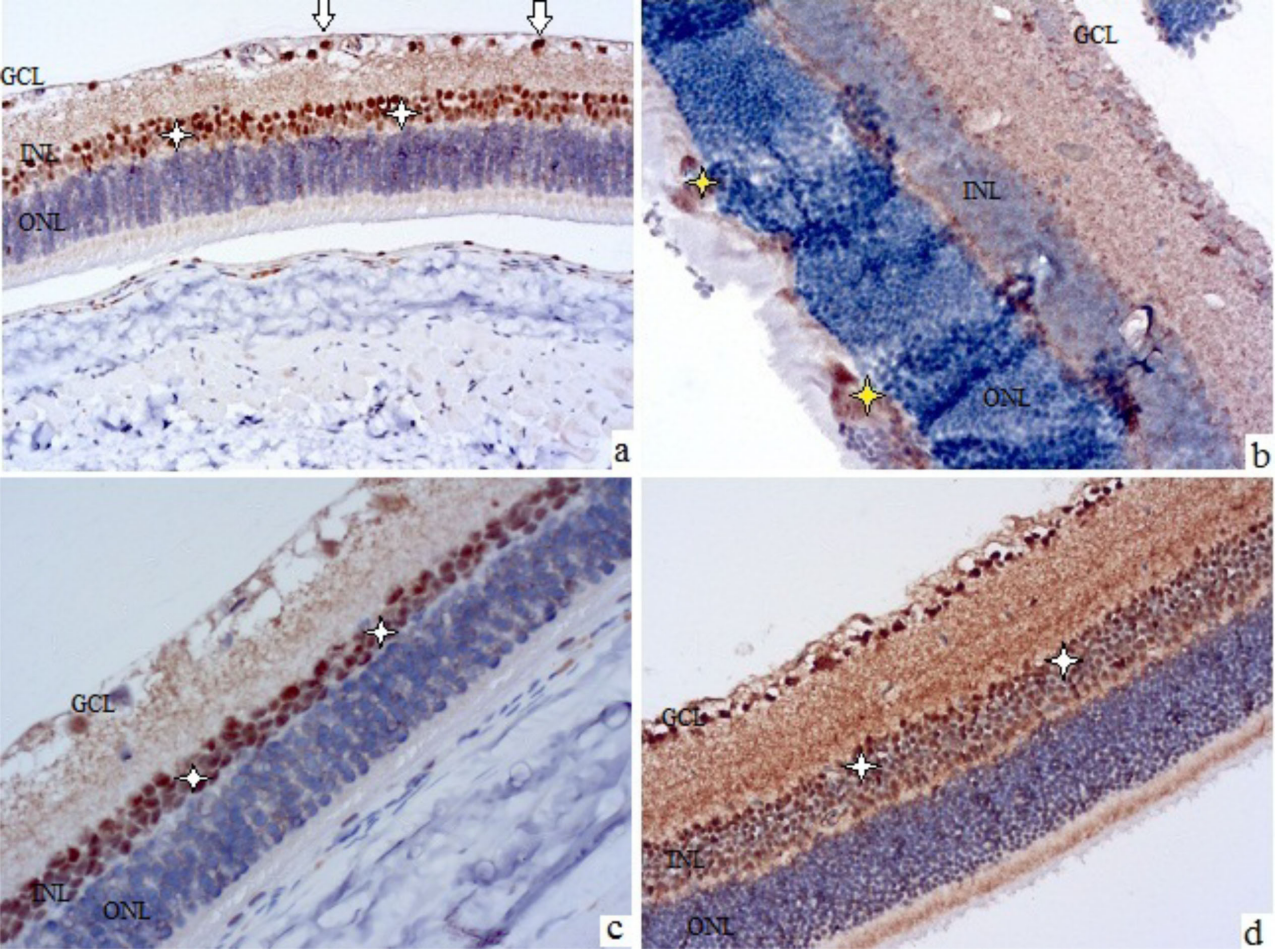
Rat retinal micropreparations at 28 days **(A, C)** and 3 months **(B, D)**. Representative results of VEGF immunohistochemical staining, counterstained with hematoxylin; ×200. GCL – ganglion cell layer, INL – inner nuclear layer, ONL – outer nuclear layer. **(A, B)** control; **(C, D)** treatment with sorafenib and insulin. In **(A, C, D)** white arrows indicate VEGF-positive cells in the nerve fiber layer, white asterisks indicate Müller cells in the inner nuclear layer; in **(B)** yellow asterisks indicate cellular proliferates in the outer nuclear layer with VEGF-positive staining elements.

**Figure 4:**
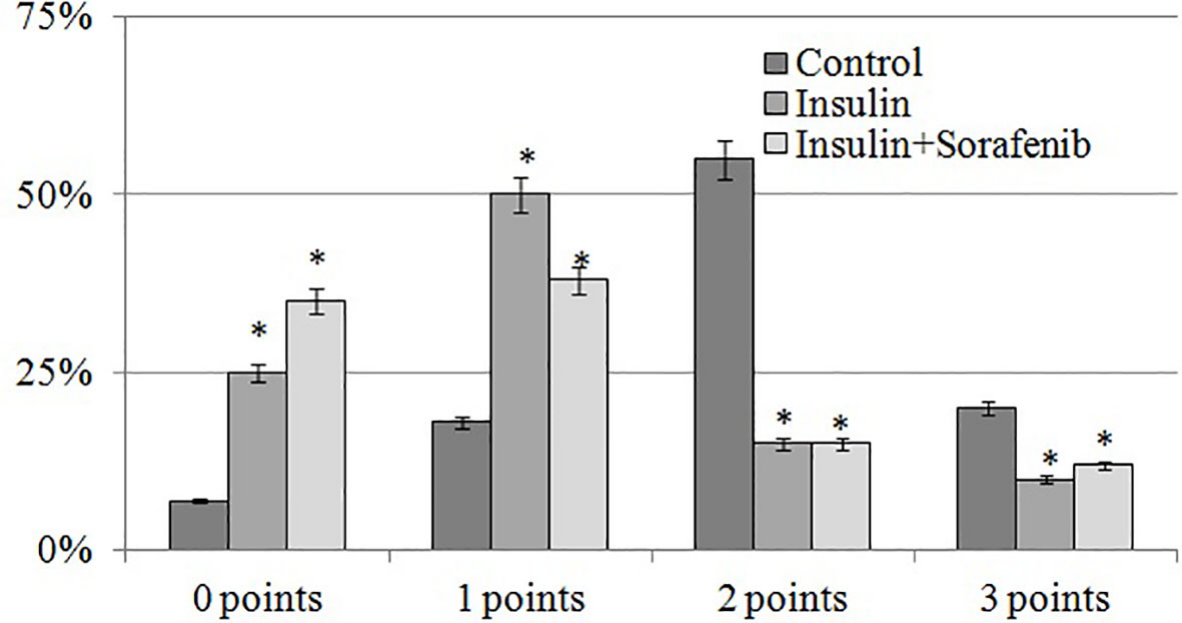
Histogram of the distribution of the number of VEGF-positive cells (%) depending on the intensity of immunospecific staining according to the scale of D.J. Dabbs at the inner nuclear layer in the 3 months; *p<0,05 compared to the control.

The results of the immunohistochemical study confirmed the upregulation of VEGF expression in retinal tissues during DR modeling (**Figures 2**-**4**). A qualitative trend toward increased intensity of VEGF-positive staining in the retina was observed at 7 and 28 days, as well as at 3 months (see **Figures 2B**, **3A, B**).

Compared with the slight staining in intact animals, which was mainly grouped in the ganglion cell layer (see **Figure 2A**), VEGF expression was much more intense after 7 days (see **Figure 2B**). VEGF-positive retinal elements could be clearly distinguished, including rounded cells located along the inner surface of the retina (see white arrows in **Figure 2B**) and small but numerous cells located along the periphery of the inner nuclear layer, probably Müller cells (see white asterisks in **Figure 2B**).

These characteristics became more evident after 28 days, when VEGF-positive astrocytes of the nerve fiber layer (see white arrows in **Figure 3A**) and Müller cells (see white asterisks in **Figure 3A**) were clearly visualized. Also, at this time, swelling and stratification of the inner layers of the retina, and the formation of foci of angiogenesis along the nerve fiber layer could be noted.

After 3 months of observation, the retinas of the control group animals (see **Figure 3B**) showed marked edema, areas of ischemia in the plexiform layers, signs of ganglion cell degeneration, and discomplexity of the layers, especially the outer nuclear layer, which contained disordered cell proliferations, some of which were VEGF-positive (see yellow asterisks in **Figure 3B**).

The use of insulin was accompanied by a retinal picture similar to that of the control group, while the combined use of sorafenib and insulin prevented the development of both morphological signs of diabetic retinopathy (DR) and an increase in the intensity of VEGF-positive staining (see **Figures 3C, D**). There were no signs of edema, ischemia, pathological angiogenesis, neurodegeneration, or layer discomplexity in the retinas of sorafenib+insulin-treated animals. Moderate VEGF-positive staining was preserved in the inner nuclear layer (white asterisks in **Figures 3C, D**) and the ganglion cell layer.

The intensity of VEGF-specific staining was most pronounced in the inner nuclear layer, and its semi-quantitative assessment using the D.J. Dabbs scale demonstrated a significant predominance of cells with strong staining (2–3 points, **Figure 4**) in the control group. Administration of insulin and insulin combined with sorafenib led to a marked increase in the proportion of unstained and weakly stained cells (0–1 point), accompanied by a decrease in the number of intensely stained cells (2–3 points). These results, in turn, confirmed the inhibitory effect of insulin and sorafenib on VEGF overexpression.

Thus, the levels of VEGF and HIF-1α in the retina were significantly elevated in experimental DR, which was attenuated by insulin administration and further prevented by the additional use of the protein kinase inhibitor sorafenib. Morphological analysis revealed the development of early DR signs associated with increased intensity of VEGF-positive staining in the retina, which was mitigated by sorafenib treatment.

## Discussion

### Key findings in the context of DR pathobiology

In the STZ-diabetes model, combined multikinase inhibition alongside insulin administration was associated with suppression of HIF1α and VEGF in the retina and preservation of retinal architecture compared with diabetic controls (**Figures 1**-**3**). These observations are consistent with the central role of the HIF1α–VEGF hypoxia–angiogenesis axis in DR and BRB dysfunction (4–6, 9–12) and align with known patterns of VEGF immunoreactivity redistribution from the ganglion cell layer/RPE under normal conditions to Müller cells, astrocytes, and ischemic regions in DR (15–20). We further focus on mechanisms, clinical relevance, and limitations.

### Pathophysiological mechanisms

Under normoxic conditions, HIF1α is hydroxylated by PHD1–3 and FIH1 (factor inhibiting HIF-1) and degraded via pVHL-dependent proteolysis; under hypoxia, it is stabilized, translocates to the nucleus, dimerizes with HIF1β, and activates transcription of VEGF and other adaptive genes (5–7, 13, 14). VEGFR2 is a key downstream mediator: phosphorylation of tyrosine Y949 (Y951 in humans) forms a TSAd (T-cell-specific adaptor)–cSrc complex, which subsequently phosphorylates VE-cadherin, disrupting inter-endothelial adhesive contacts. Phosphorylation of tight junction proteins (claudins, occludin, ZO-1) further increases BRB permeability (9–11, 21–23). Pericytes stabilize microvessels and the BRB, and their dysfunction exacerbates microangiopathy (12, 46–49). At the neurogliovascular unit level, NO (nitric oxide)/glutamate-mediated pathways (NOGC/cGMP/PKG; NO-sensitive guanylate cyclases/cyclic GMP/protein kinase G) and impaired glutamate transport contribute to neurodistress and may act synergistically with VEGF-mediated barrier dysfunction (43, 50–54). This background directly supports the interpretation of HIF1α/VEGF suppression observed following kinase blockade in our model.

### Hyperglycemia- and hypoxia-induced upregulation of HIF1α/VEGF and their modulation by insulin

Chronic hyperglycemia creates a pro-angiogenic microenvironment through inflammation (IL1β/TNFα/IL18/IL17A – interleukin-1β/tumor necrosis factor-α/interleukin-18/interleukin-17A, NFκB – nuclear factor kappa B-dependent mechanisms), oxidative stress (polyol pathway, hexosamine pathway, PKCβ – protein kinase Cβ activation), the AGEs-RAGE axis (advanced glycation end-products and their receptors), as well as GPR91 (G protein-coupled receptor 91)–MAPK/COX2 (cyclooxygenase-2)/PGE2 (prostaglandin E2) (44, 45, 55–63). Early microvascular events (leukostasis, vasoconstriction) exacerbate hypoperfusion and stabilize HIF1α, increasing VEGF and NOS levels; in ocular tissues, HIF1α rises earlier than VEGF and correlates with DR progression (44, 45, 64, 65). Additionally, PKC/MAPK, PI3K/Akt/mTOR (phosphoinositide 3-kinase/protein kinase B/mTOR), and NFκB enhance HIF1α transcription/translation, further amplifying VEGF signaling (66–70).

On this background, the effect of insulin is ambiguous: in clinical settings, early worsening of DR and increased risk of macular edema may occur with rapid glycemic improvement (24–26), while in retinal endothelium, insulin and high glucose exert non-additive and dose-dependent opposing effects on VEGF/ROS (reactive oxygen species) (71, 72). The involvement of NOX4 (an NADPH oxidase isoform) in the HIF1α/VEGF response, as well as variability in genetic determinants of VEGF/VEGFR, may explain individual differences (73, 74). In our experiment, this is reflected in the heterogeneity of VEGF response to insulin and explains why monotherapy targeting metabolic control only partially affects HIF1α/VEGF.

### Mechanisms and sites of action of sorafenib (multi-kinase blockade)

Sorafenib acts on two levels. Upstream: inhibition of cRAF/BRAF disrupts the MAPK/ERK node, which supports HIF1α-dependent VEGF expression and expansion of the angiogenic transcriptome (27, 30, 75). Receptor level:blockade of VEGFR1/2/3 and PDGFRβ suppresses post-receptor events (permeability, endothelial proliferation, neo-(lymph)angiogenesis, and pericyte recruitment) (9–12, 21–23, 27–30, 75–77). Additionally, suppression of HIF1α translation via mTOR/RPS6KB1/4EBP1 without altering mRNA levels, as well as interference with nuclear translocation/binding of HIF1α to HRE sites, has been described (28, 29, 31). Collectively, these mechanisms logically explain the attenuation of the hypoxia-driven angiogenic response in our model. We do not ascribe a direct antioxidant effect to the drug; the potential reduction in ROS/HIF1α stabilization is considered secondary to inhibition of RAF/MAPK/VEGFR (78). At the microvascular level, PDGFRβ-mediated pericyte recruitment and stabilization of newly formed vessels may also be suppressed (46, 48), whereas VEGFR3 is involved in pathological neo-(lymph)angiogenesis (77). This “multi-node” effect is consistent with normalization of VEGF/HIF1α localization and preservation of retinal layer organization in the insulin+sorafenib group.

### Analytical and methodological aspects relevant for interpretation

The study design included time points at 7, 28 days, and 3 months, allowing the tracking of transitions from early molecular events to morphological features of diabetic retinopathy (DR); this approach is consistent with data showing a preceding increase in HIF1α levels compared to VEGF during retinal ischemia (64). Quantitative assessment of VEGF was performed by Western blot under reducing and non-reducing conditions to distinguish the monomer (~26 kDa) from the disulfide-linked dimer (~45 kDa), while immunohistochemistry confirmed the topographic expansion of VEGF-positive cells (ganglion cell layer, Müller cells, astrocytes, ischemic zones) in control DR, with a partial reversal following sorafenib treatment (15–20). The combination of these approaches minimizes the risk of misinterpretation from a single method and strengthens causal linkage to the HIF1α–VEGF axis.

### Clinical relevance and safety

Intravitreal anti-VEGF therapy is the standard for macular edema and neovascularization; however, response variability, injection burden, and the persistence of upstream drivers remain challenges (9–12, 21–23). Our data support the concept of complementarity: multikinase blockade can reduce HIF1α-dependent pathways and VEGFR/PDGFR signaling, theoretically complementing anti-VEGF therapy in scenarios of residual hypoxia or high injection frequency (9–12, 21–23, 27–31, 70, 75, 76, 78, 79). At the same time, we do not propose systemic sorafenib as a ready treatment for DR: safety limitations for the multikinase inhibitor class upon systemic administration are well documented (dermatologic, cardiovascular, neurological, and other adverse effects; class-specific effects in oncology) (32–34). Therefore, translationally more feasible approaches appear to be local delivery modes (intraocular microdoses/depot formulations) or more selective agents targeting RAF/MAPK or VEGFR/PDGFR with minimal systemic impact (11, 27, 30, 76). Additionally, in the eye, prolonged PDGFRβ blockade should be avoided to prevent disruption of pericyte-mediated stabilization of newly formed vessels (46, 48).

### Autophagy

The role of autophagy in microvascular remodeling in diabetes is actively discussed, particularly regarding interactions with HIF1α, mTOR, and the Bnip3/FoxO3a axis (80–84). We did not experimentally assess these pathways, and therefore refrain from causal claims, presenting them only as a potential modulatory context warranting further investigation.

## Limitations and future directions

Animal sex. Studies were conducted on males to minimize hormonal variability and estrogen-dependent modulation of VEGF/HIF1α and BRB permeability; extrapolation to females requires validation with estrous stratification and hormonal profiling (11, 35–38).

Observation period. The study covered early-to-intermediate time points, so late-stage DR and long-term intervention outcomes were not assessed.

Functional endpoints. ERG and behavioral vision assessments were not included; bridging molecular/morphological effects to visual function requires further validation.

Potential off-target effects. Multikinase inhibition may have non-retinal consequences; given the class profile, systemic sorafenib for DR is limited, whereas local or more selective approaches appear more promising (11, 27–30, 75).

Future directions naturally arise from these limitations: inclusion of sex as a variable—replication in females with estrous stratification and hormonal profiling; extended observation periods to assess effect durability and interactions with late-stage DR; functional validation (ERG, optokinetic reflex, behavioral assays) to link HIF1α/VEGF dynamics and morphology to visual function; and delivery/selectivity platforms—testing local regimens (intravitreal, microdosing) and/or more selective inhibitors of upstream/receptor nodes as potentially safer alternatives to systemic multikinase blockade (9–12, 21–23, 27–30, 75–77, 85). This mechanistically and clinically oriented approach aligns with contemporary DR management strategies and may reduce injection burden and instances of anti-VEGF monotherapy resistance.

## Concluding remarks and translational outlook

Our data support the notion that the HIF1α–VEGF axis is central to early hypoxia-driven angiogenic changes in DR. Insulin partially attenuates this activity but does not fully normalize it, whereas combining insulin with multikinase blockade prevents VEGF upregulation and topographical shifts, preserving retinal architecture over the studied period. Mechanistically, these effects are consistent with upstream (RAF/MAPK) and receptor-level (VEGFR/PDGFR) actions of sorafenib, reinforcing the rationale for complementarity with anti-VEGF strategies. Translationally, local or more selective targeting approaches appear promising, provided careful safety evaluation. Our study was male-only, short-term, and lacked functional visual metrics (ERG), highlighting the need for studies in females, extended observation periods, and functional validation before clinical translation.

## Data Availability

The original contributions presented in the study are included in the article/supplementary material. Further inquiries can be directed to the corresponding author.
